# Difference in the anterior displacement of the tibial tuberosity relative to the proximal tibial fragment between opening wedge and closed wedge high tibial osteotomies

**DOI:** 10.1051/sicotj/2024020

**Published:** 2024-05-30

**Authors:** Kentaro Kikuchi, Ken Kumagai, Shunsuke Yamada, Shuntaro Nejima, Hyonmin Choe, Hiroyuki Ike, Naomi Kobayashi, Yutaka Inaba

**Affiliations:** 1 Department of Orthopaedic Surgery, Graduate School of Medicine, Yokohama City University Yokohama 236-0004 Japan; 2 Department of Orthopaedic Surgery, Yokohama City University Medical Center Yokohama 232-0024 Japan

**Keywords:** High tibial osteotomy, Closed wedge, Opening wedge, Tibial tuberosity, Anterior displacement

## Abstract

*Introduction*: This study aimed to investigate the anterior-posterior (AP) displacement of the tibial tuberosity (TT) and to assess the difference between closed wedge and opening wedge high tibial osteotomies (OWHTO and CWHTO). *Methods*: One hundred consecutive knees with osteoarthritis that underwent OWHTO (50 knees) or CWHTO (50 knees) were investigated retrospectively. The femorotibial angle (FTA) was measured on AP radiographs of the knee. AP displacement of the TT, posterior tibial slope (PTS), the modified Blackburne-Peel index (mBPI), and the modified Caton-Deschamps index (mCDI) were measured on lateral radiographs of the knee. *Results*: Patients had a mean correction angle of 12.58 ± 2.84° and 18.98 ± 5.14° (*P* < 0.001), with a mean AP displacement of TT of 0.84 ± 2.66 mm and 7.78 ± 3.41 mm (*P* < 0.001) in OWHTO and CWHTO, respectively. The AP displacement of the TT per correction of 1° was significantly greater in CWHTO than in OWHTO (*P* < 0.001). A significant correlation was found between the correction angle and AP displacement of the TT in CWHTO (*r* = −0.523, *P* < 0.001), but not in OWHTO. The change of PTS per correction of 1° was significantly greater in OWHTO than in CWHTO (*P* < 0.001). The changes of mBPI and mCDI per correction of 1° were significantly greater in CWHTO than in OWHTO (*P* < 0.001 and *P* < 0.001, respectively). *Conclusions*: There was greater anterior displacement of the TT in CWHTO than in OWHTO, which was correlated with the correction angle. The results suggested that CWHTO would be better than OWHTO when a concomitant anteriorization of TT is required.

## Introduction

High tibial osteotomy (HTO) is a joint-preserving surgical procedure, utilized to address medial compartmental osteoarthritis (OA) of the knee. Two procedures closed wedge and opening wedge HTOs (CWHTO and OWHTO), are commonly used, and outstanding clinical results have been documented. HTO is designed to address coronal malalignment and minimize mechanical stress on the affected compartment of the knee. This basic concept of change in the weight-bearing axis offers relief from pain and subsequently enhances knee function [1, 2]. In addition to the change of coronal alignment, unintended change in sagittal alignment can potentially occur.

An unintended alteration in the sagittal plane is seen as a discrepancy of the axes between the proximal and distal tibial fragments after osteotomy. For example, the postoperative change of the posterior tibial slope (PTS) is a well-known unintended alteration in the sagittal plane, which is seen as an increase after OWHTO and a decrease after CWHTO [3]. The proximal or distal displacement of the tibial tuberosity (TT) after HTO is also an unintended change in the sagittal plane, which is related to the occurrence of patella alta or infera [4, 5]. These changes could affect congruity and contact pressure in the patellofemoral (PF) joint [6–8]. The anterior-posterior (AP) displacement of the osteotomized fragment is thought to be another unintended change in the sagittal plane, which also potentially affects the PF compartment. It is assumed that the TT is more anteriorly displaced in CWHTO than in OWHTO since the distal fragment in CWHTO is moved forward anteriorly for the flange thickness at the TT, as well as proximally. However, the magnitude of the postoperative change in this direction and the difference between CWHTO and OWHTO have not been well documented. This study focused on the AP displacement of the TT in both CWHTO and OWHTO.

The focus of this study was to explore the AP displacement of the TT in the sagittal plane and to assess the difference between CWHTO and OWHTO. We hypothesized that the anterior displacement of the TT is greater in CWHTO compared to OWHTO.

## Materials and methods

### Patients

A retrospective investigation was conducted on a total of 100 consecutive knees belonging to 86 patients with knee OA who underwent either OWHTO between 2014 and 2017 or CWHTO between 2012 and 2016. The eligibility requirement in this study involved individuals with painful OA specifically affecting the medial compartment of the knee. Those ineligible had OA in the lateral compartment, a flexion contracture exceeding 15°, or a medical history involving inflammatory arthritis such as rheumatoid arthritis. The HTO technique was determined based on the correction angle. OWHTO was carried out on 50 knees of 45 patients with an angular correction of 15° or below, and CWHTO was carried out on 50 knees of 41 patients with an angular correction exceeding 15°. Table 1 shows the demographic data. Our institutional review board granted approval for the study protocol and publication. Each participant included in the study provided written informed consent.

**Table 1:** Demographic data

	OWHTO	CWHTO	*P* value
	(*n* = 50)	(*n* = 50)	
Male/female	17/33	14/36	0.527
Age (year)*	65.6 ± 9.6	66.7 ± 9.2	0.538
FTA (°)*	181.4 ± 2.75	187.2 ± 3.23	< 0.001
Radiographic classification^a^: Grade 1/2/3/4/5	0/11/28/11/0	0/4/25/21/0	0.038

* The values are given as mean ± standard deviation.

a OA grade modified from Ahlbäck classification.

OWHTO: opening wedge high tibial osteotomy, CWHTO: closed wedge high tibial osteotomy, FTA: femoral tibial angle.

### Surgical procedure and postoperative management

The preoperative planning involved determining the angular correction with achieving a valgus of 10° in anatomical tibiofemoral angle for a standing position since favorable long-term clinical results are anticipated by achieving this target angle [2, 9, 10]. The angular correction is restricted to 15° or lower in OWHTO. In contrast, a greater correction is feasible in CWHTO. The choice between the two techniques was determined preoperatively in accordance with the angular correction, following our institutional guideline [10–12]. Specifically, OWHTO was carried out in knees with an angular correction of 15° or less, while CWHTO was chosen for knees with an angular correction exceeding 15°.

OWHTO was conducted from an anteromedial side of the tibia using fluoroscopy. Using a biplanar technique, a diagonal osteotomy was executed, starting from the medial cortex at 35 mm below the medial articular surface, and extending to the upper part of the proximal tibiofibular joint, while preserving the TT. The gap between osteotomy surfaces was widened and two wedge-shaped β-TCP blocks were placed. The fixation was accomplished using TomoFix.

CWHTO was conducted from an anterolateral side of the tibia using fluoroscopy, following 10–20 mm segmental osteotomy of the fibula. The proximal osteotomy was executed parallel to the tibial plateau, initiating 30 mm below the lateral articular surface of the tibia. The distal osteotomy was executed in an oblique manner, directed to the medial cortex hinge point. The patellar tendon insertion was retained incorporating a distal fragment as a flange. The tibial fragments were secured using an OWL plate after closing the osteotomy gap.

Following surgery, patients initiated a rehabilitation protocol starting the day after the procedure. Full weight-bearing was allowed 3 weeks after surgery in CWHTO and one week after surgery in OWHTO.

### Radiographic measurements

**Figure 1:**
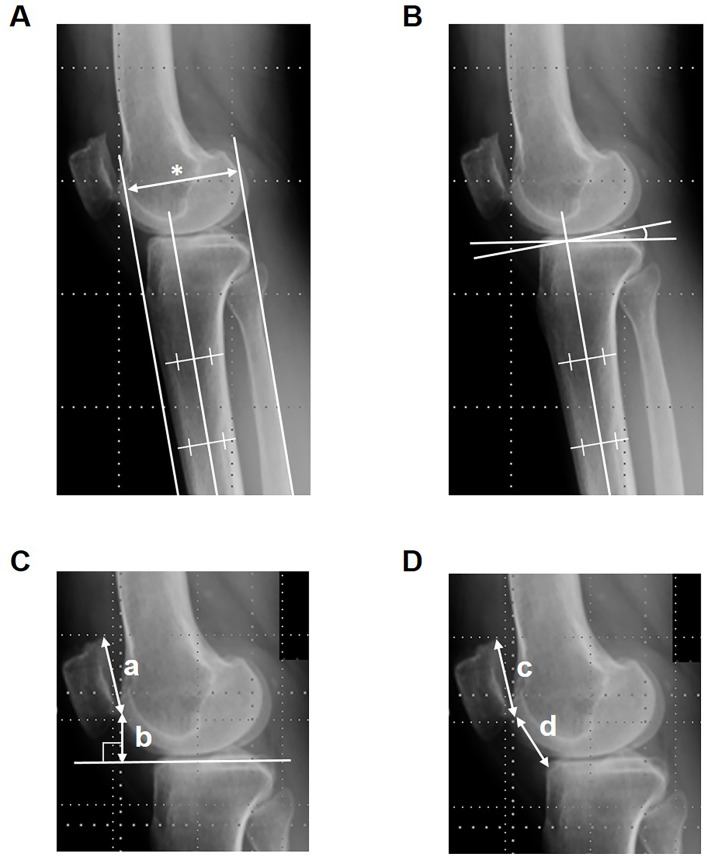
Radiographic measurements. (A) Distance between the anterior border of the tibial tuberosity and the posterior border of the medial tibial condyle (*). (B) Posterior tibial slope. (C) Modified Blackburne-Peel index (b/a). (D) Modified Caton-Deschamps index (d/c).

Two orthopedic surgeons independently conducted the measurements in radiographs, and the mean values of the measurements taken by the two surgeons were used in the analysis. The AP radiographs were taken to confirm a centered position of the patella between the femoral condyles, and the lateral radiographs were taken to confirm adequate overlapping of the posterior sections of the medial and lateral condyles, clear visibility of the patella with a distinct patellofemoral joint space, and minimal overlapping of the tibial and femoral platforms. The femorotibial angle (FTA) was measured using AP radiographs of the knee in a standing position. Using lateral radiographs of the knee, tangent lines to the anterior border of the TT and the posterior border of the medial tibial condyle were drawn perpendicular to the tibial shaft axis. The distance between these two lines was measured, and the amount of change was defined as the AP displacement of the TT (Figure 1a). The posterior tibial slope (PTS) (Figure 1b), the modified Blackburne-Peel index (mBPI) [4, 13] (Figure 1c), and the modified Caton-Deschamps index (mCDI) [14, 15] (Figure 1d) were also measured using lateral radiographs of the knee. All radiographic images were acquired digitally. The amounts of the changes from preoperative to postoperative in the FTA, PTS, mBPI, and mCDI were defined as ∆FTA, ∆PTS, ∆mBPI, and ∆mCDI, respectively.

### Statistical analysis

The statistical analysis was conducted using BellCurve for Excel version 4.02 (Social Survey Research Information Co., Ltd., Tokyo, Japan). Due to non-normal distributions observed in the histograms of the data, nonparametric statistical methods were employed for analysis. The Mann–Whitney *U* test was utilized to assess significant differences in continuous variables, while Pearson’s chi-squared test was applied to examine significant differences in the distributions of categorical variables. Spearman’s rank correlation coefficient was utilized to identify relationships between two variables, and was interpreted as follows; negligible (0–0.09), weak (0.1–0.39), moderate (0.4–0.69), strong (0.7–0.89), very strong (0.9–1) [16]. An adjusted *p*-value below 0.05 was considered statistically significant. Intraclass correlation coefficients (ICCs) were computed to assess the reliability of radiographic measurements.

## Results

**Figure 2:**
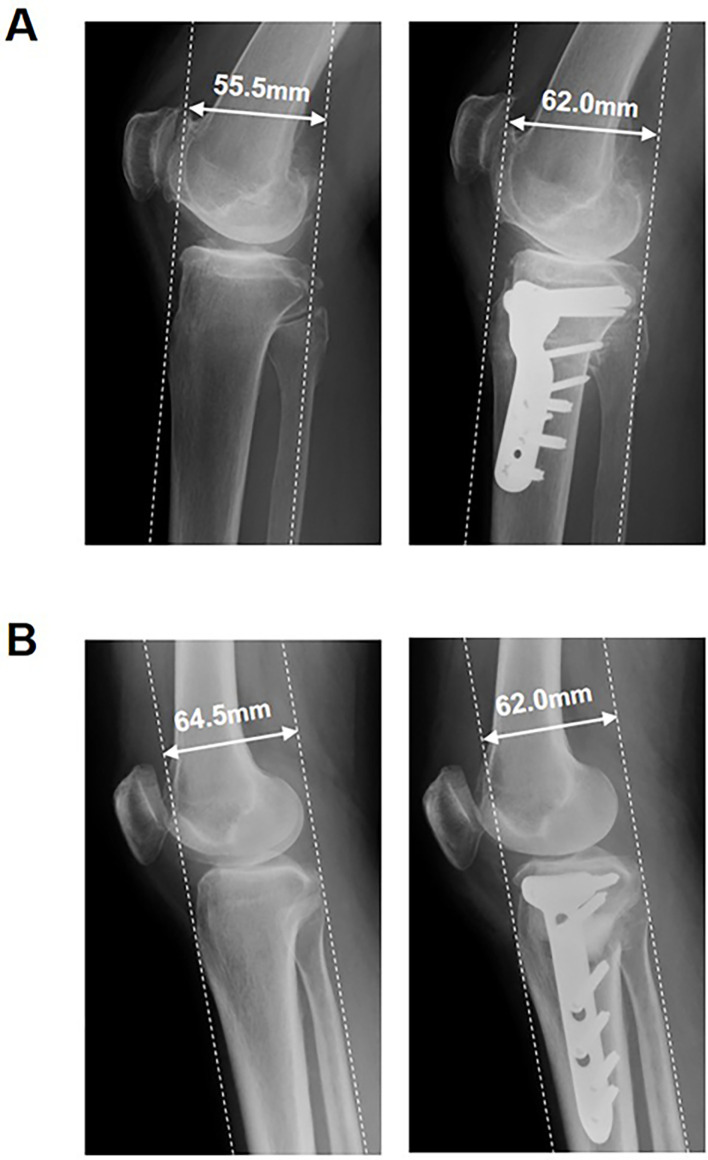
Pre- and postoperative radiographs from one patient each who underwent closed wedge high tibial osteotomy (A) and opening wedge high tibial osteotomy (B). (A) The distance between the anterior border of the tibial tuberosity and the posterior border of the medial tibial condyle is 55.5 mm preoperatively (left) and 62.0 mm postoperatively (right), indicating an increase of 6.5 mm. (B) The distance between the anterior border of the tibial tuberosity and the posterior border of the medial tibial condyle is 64.5 mm preoperatively (left) and 62.0 mm postoperatively (right), indicating a decrease of 2.5 mm.

The mean changes in radiographic parameters are summarized in Table 2. Patients had a mean correction angle of 12.58 ± 2.84° and 18.98 ± 5.14° (*P* < 0.001), with a mean AP displacement of TT of 0.84 ± 2.66 mm and 7.78 ± 3.41 mm (*P* < 0.001) in OWHTO and CWHTO, respectively. Representative radiographs are shown in Figure 2. The PTS increased by 3.62 ± 1.97° in OWHTO and decreased by 2.89 ± 2.03° in CWHTO (*P* < 0.001). The mBPI and mCDI decreased by 0.20 ± 0.13 and 0.22 ± 0.16 in OWHTO and increased by 0.01 ± 0.18 and 0.03 ± 0.19 in CWHTO (*P* < 0.001 and *P* < 0.001), respectively. The mean changes in radiographic parameters per correction angle are summarized in Table 3. The AP displacement of the TT per correction of 1° was significantly greater in CWHTO than in OWHTO (*P* < 0.001). The ∆PTS per correction of 1° was significantly greater in OWHTO than in CWHTO (*P* < 0.001). The ∆mBPI and ∆mCDI per correction of 1° were significantly greater in CWHTO than in OWHTO (*P* < 0.001 and *P* < 0.001, respectively). The ICCs for inter-rater and intra-rater reliabilities for radiographic measurements were all >0.8, ranging from 0.86 to 0.98, indicating good reliability.

**Table 2:** Mean changes of radiographic parameters.

	OWHTO	CWHTO	*P* value
AP displacement of TT (mm)	0.85 ± 2.66	7.78 ± 3.41	<0.001
∆FTA (°)	12.58 ± 2.84	18.98 ± 5.14	<0.001
∆PTS (°)	3.62 ± 1.97	−2.89 ± 2.03	<0.001
∆mBPI	−0.20 ± 0.13	0.01 ± 0.18	<0.001
∆mCDI	−0.22 ± 0.16	0.03 ± 0.19	<0.001

The values are given as mean ± standard deviation.

AP: anterior-posterior, TT: tibial tuberosity, FTA: femoral tibial angle, PTS: posterior tibial slope, mBPI: modified Blackburne-Peel index, mCDI: modified Caton-Deschamps index.

**Table 3:** Mean changes of radiographic parameters per correction angle.

	OWHTO	CWHTO	*P* value
AP displacement of TT/∆FTA (mm)	0.0674 ± 0.2116	0.4099 ± 0.1795	<0.001
∆PTS/∆FTA (°)	0.2876 ± 0.1565	−0.1522 ± 0.1070	<0.001
∆mBPI/∆FTA	−0.0162 ± 0.0106	0.0005 ± 0.009	<0.001
∆mCDI/∆FTA	−0.0174 ± 0.0129	0.0018 ± 0.0099	<0.001

The values are given as mean ± standard deviation.

AP: anterior–posterior, TT: tibial tuberosity, FTA: femoral tibial angle, PTS: posterior tibial slope, mBPI: modified Blackburne-Peel index, mCDI: modified Caton-Deschamps index.

**Figure 3:**
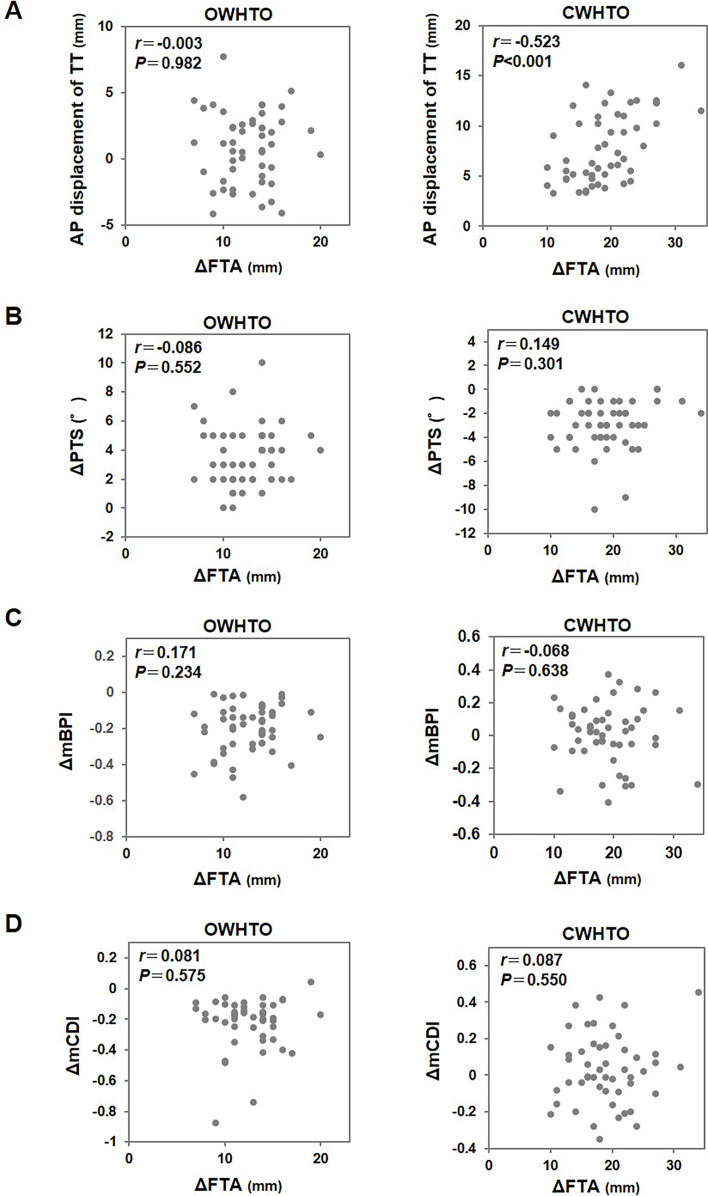
Scatter plots showing the relationships between the correction angle (∆FTA) and radiographic parameters. (A) anterior-posterior displacement of the tibial tuberosity, (B) change of posterior tibial slope (∆PTS), (C) change of the modified Blackburne-Peel index (∆mBPI), and (D) change of the modified Caton-Deschamps index (∆mCDI) in OWHTO (left panels) and CWHTO (right panels).

To assess the correction angle and radiographic parameters, ∆FTA was plotted against AP displacement of the TT, ∆PTS, ∆BPI, or ∆CDI in each HTO procedure (Figure 3). A significant correlation was found between ∆FTA and AP displacement of the TT in CWHTO (*r* = −0.523, *P* < 0.001), but not OWHTO. There were no significant correlations between ∆FTA and ∆PTS, ∆BPI, or ∆CDI in both HTO procedures.

## Discussion

The main findings of this study were that (1) CWHTO resulted in greater anterior displacement of the TT than OWHTO, and (2) anterior displacement of the TT was correlated with the correction angle in CWHTO. These results supported the initial hypothesis.

Unintended changes in radiographic parameters in the sagittal plane have often been discussed, with differences between OWHTO and CWHTO [17–19]. A meta-analysis showed that OWHTO increases the PTS and decreases the patellar height, whereas CWHTO leads to a decrease in the PTS and no change in the patellar height [3]. Since a reduced patellar height is associated with an elevated PF contact pressure [7, 8], the PF joint would be more frequently affected by OWHTO than CWHTO [20]. An increase in PTS leads to anterior translation of the tibia and increases tension in the ACL [21], whereas decreased PTS leads to posterior translation of the tibia and increases tension in the PCL [22]. In addition to these changes in the sagittal plane, a few studies showed a change of the osteotomized fragment in the AP direction after HTO, i.e. in OWHTO, the TT is not only moved to the distal direction but also moved to the posterior direction in relation to the biplane osteotomy [23]. No comparative study has been conducted to assess changes in the AP direction of the distal fragment in relation to the proximal fragment between OWHTO and CWHTO, and the present study focused on it.

There are no commonly accepted parameters to assess the relative AP position of the fragments. In the present study, the AP displacement of the osteotomized fragment was defined as the postoperative change of the relative position of the TT to the proximal fragment, based on the measurements of the distance between the tibial tubercle prominence and the posterior border of the medial tibial condyle. Zheng et al. investigated the change of relative AP position in two osteotomized fragments of OWHTO by measuring the vertical distance from the most prominent point of the tibial tubercle to the perpendicular line of the tangent of the medial tibial plateau, and the biplane ascending osteotomy showed more rearward movement of the TT from the proximal fragment than the uniplane osteotomy [23]. Although they reported good reproducibility of the measuring method, it may be potentially affected by the change in PTS. Therefore, in the present study, the relative AP displacement of the fragment was defined by the measurement that was not affected by PTS. Since this measuring method was also highly reproducible (ICC > 0.8), this method seems reliable for assessing the AP displacement of the fragment.

Increased contact pressure potentially leads to the progression of OA in the affected compartment. For isolated PF-OA, anterior advancement of the TT has been applied as the procedure to reduce mechanical stress on the PF compartment [24, 25]. A cadaveric study demonstrated that trochlear contact force was significantly decreased by 1 cm straight anteriorization of the TT [26]. For combined medial and PF compartmental OA, a concomitant procedure of HTO and anteriorization of the TT has been attempted to reduce the bicompartmental mechanical load on the knee joint, resulting in good clinical outcomes [27]. This study demonstrated an average anterior displacement of the tibial tuberosity of 7 mm after isolated CWHTO, although anteriorization of the distal fragment was not intended. CWHTO may have the potential to reduce the contact force on the PF joint.

Several studies reported the effect of HTO on the cartilage status of the PF compartment, especially highlighted in OWHTO [29–31]. OWHTO decreases patellar height, which is related to an increase in the contact force in the PF compartment [7, 8]. In addition, patellar tracking and PF congruity are worse in OWHTO than in CWHTO [6, 32]. The present study demonstrated that CWHTO did not affect patellar height and advanced the TT anteriorly compared to OWHTO, suggesting that CWHTO would be a better choice in cases with degenerative changes of the PF compartment.

This study has several limitations. First, this was a retrospective, nonrandomized, sequential radiographic review. Second, preoperative OA grade and correction angle differed between the two HTO procedures. Thus, radiographic parameters per correction angle were also assessed. Third, there was no data regarding clinical outcomes such as patient-reported outcome measures, and whether differences in sagittal changes between the two HTO procedures affect clinical outcomes is unknown. Fourth, the radiographs were not calibrated using reference markers.

## Conclusion

CWHTO showed a greater anterior displacement of the TT than OWHTO, which was correlated with the correction angle. These results suggest that CWHTO would be better than OWHTO when a concomitant anteriorization of TT is required.

## Data Availability

Available upon request from the corresponding author.
